# Comparative Values of the Different Silicate Cements

**Published:** 1910-12-15

**Authors:** W. B. Cavanagh

**Affiliations:** Cornwall, Ontario


### COMPARATIVE VALUES OF THE DIFFERENT SILICATE CEMENTS.

W. B. CAVANAGH, CORNWALL, ONTARIO.

Read before the Eastern Ontario Dental Association.

Since the intoduction of plastic cements for filling cavities in the natural teeth all efforts of chemists have been directed towards the production of a durable preparation which would more or less resemble the natural organs in appearance. The initial efforts of utilizing silicate mixtures for this purpose were made many years ago, but, like many other first attempts, they were not an unqualified success. It was recognized that the silicate preparations possessed a degree of translucency which made them of inestimable value in certain cavities exposed to view. Its chief properties over other cements used as filling materials are: (a) Its extreme hardness; (b) its great resistance to the destructive action to the oral fluids; (c) strength or resistance to wear; (d) its close resemblance to the natural enamel.

I shall now give the results of a few of the investigations made by chemists into the properties of silicate cements. Their investigations cover the properties known as: (1) Strength, or resistance to wear; (2) Adhesion; (3) Alteration in bulk, color or surface, and (4) Resistance to the oral fluids.

1. *Strength.*—In lbs. per sq. m.m. made with bars $4\frac{1}{2}$ m.m. sq.; fifteen tests. Average taken. Aschers, 1.17; Astral, 1.12; Harvadid, 0.95; Schoenbecks, 1.21.

2. *Adhesion.*—Average in lbs. Six tests made with same tooth. Natural teeth were taken and the crowns ground down level to full exposure of the dentine, the cements were mixed and built upon these teeth with a loop of wire embedded in the cement. After two days the hook of a spring-balance was inserted through the loop of wire and the number of lbs. was recorded when the cement broke away from the tooth. Aschers, 0.58 lbs.; Astral, 3.70 lbs.; Harvadid, 3.08 lbs.; Schoenbecks, 20.66 lbs.

Silicates adhere more to dentine than to enamel, and for this reason the practice of covering all dentine in a cavity with other materials, under silicate fillings, deprives the silicate of some of its adhesive property. It also impairs to some extent its translucency.

3. *Shrinkage.*—Out of the mouth. Six tests. Average loss for each 100 grs. after standing three days:—Aschers, 2.34 m.m.; Astral, 2.38 m.m.; Harvadid, 3.06 m.m.; Schoenbecks, 0.99 m.m.

4. *Loss of Weight.*—Out of mouth. Six tests for each 100 grs., three days after mixing. Aschers, 2.52 grs.; Astral, 1.70 grs.; Harvadid, 2.36 grs.; Schoenbecks, 0.89 grs.

5. Variations in "bulk" and weight under mouth conditions:

Variations in bulk is stated in m.m. per 100 grs. weight.

Variations in weight is stated in grs. per 100 grs., one hour after mixing. Material varnished and placed in warm water for three days and then removed.

| | Aschers. | Astral. | Harvadid. | Schoenbecks. |
|---|---|---|---|---|
| Bulk | +0.55 | +0.75 | +0.78 | +0.29 |
| Weight | +0.50 | +0.60 | +0.58 | +2.19 |

It is difficult to account for the increase in weight in each case unless the coating of varnish fails to be entirely impervious to moisture. That varnishing minimizes the absorption of water is proved by making the same tests without varnishing the mixture. This test also shows that shrinkage is governed by loss of weight.

The foregoing shows that in the mouth Aschers, Astral and Harvadid shows a slight shrinkage, while Schoenbecks shows an expansion as it is a great absorbent of water, but this can be greatly reduced by the use of a good varnish.

By further investigations into the action of various liquids on these cements, the following deductions were made: Water solution of sodium chloride, sodium carbonate, alum, alcohol and ether are about equally absorbed by each cement. Astral absorbs the least; Schoenbecks the most, which is its greatest defect.

Alum has a great effect upon the cements, producing deep fissures and cracks; the other solutions do not appear to effect the surfaces excepting the fact that any watery solution coming in contact with these cements in the early stages of crystallization decolorizes the surface and reduces it to a loose, light powder. For that reason it is detrimental to wash silicate fillings with alcohol before coating with varnish.

A mineral acid, such as sulphuric, even a weak solution, has a very destructive action upon these cements, especially Aschers, which mixes much coarser than the others, and probably for this reason is more readily acted upon. Diluted sulphuric in two days reduces Aschers cement 50 per cent. in weight. Astral cement seems to resist such an acid the best.

Carbolic tooth powder used where any bleeding of the gums occurs will produce, in as short a period as three months, very dark staining of silicate cement fillings. This was recorded from a case where the fillings had remained perfect for eighteen months previous to the use of this powder.

As the exclusion of external moisture during the period of setting of silicate cements seemed to be of extreme importance I record the facts of "dry heat" during the setting. By the application of dry heat to 100 deg. F. setting takes place in one-sixth of the usual time, but translucency is deteriorated, especially with Aschers and Harvadid. Dry heat will also accentuate any shrinkage.

The rationale of this experiment is that internal moisture (water of crystalization) is requisite for the best results to be obtained, and that it is detrimental to success to use dry heat, as it has been shown to permit saliva or external moisture to come into contact with silicates during the period of setting exposure to air does not appear to effect the translucency to any extent.

Summary of the various points concerning each cement:

(a) Period of setting is in the following order—Aschers, Harvadid, Astral, Schoenbecks.

(b) Plasticity and adhesion—Schoenbecks, Astral, Harvadid, Aschers.

(c) Alterations in bulk, color, and resistance to oral fluids after proper protection during the setting period.

(1) Bulk.—Schoenbecks, Aschers, Harvadid, Astral.

(2) Resistance of fluids of mouth.—Astral first, others about equal.

(3) Colorer.—All good with exception of chalky white spots in Aschers, probably due to the coarseness of the powder.

(4) Translucency and surface gloss is very good with Astral and Schoenbecks, but not so good with Aschers and Harvadid.

### ESSENTIALS FOR SUCCESS WITH SILICATE FILLINGS.

Mixing slab, spatula, and instruments to be scupulously clean and kept for this use exclusively. They should be of agate.

Mixing the cement should be done as quickly as possible and at first by "stirring" continuously more and more powder into the liquid, then complete the process by spatulating further powder into the liquid, spreading it over as small a surface as possible. This should be continued until the previous greasy surface of the cement disappears; it should now be the consistency of soft butter. Now mould the mass upon the edge of the slab and condense the cement with the spatula until the greasy surface disappears.

The cement should be quickly inserted in the tooth in small portions, attending specially to any cervical margin, groove or undercut. All excess of cement should be avoided; obtain shape and contour before the cement begins to set. This gives natural setting, gloss and translucency. Avoid any friction after cement has begun to set as this produces heat and causes loss of water of crystallization, which spoils the translucency of the cement. Patting the surface of the cement with agate points smeared with cocoa butter brings out the best results in gloss and translucency. Use a celluloid strip matrix smeared with cocoa butter to press the cement firmly into the cavity, cutting away from margin of cavity all excess. When cement has set sufficiently apply four coats of thin varnish, giving each ample time to dry and harden.

It is necessary to take great care with the liquid supplied with silicate cements. Avoid any contamination of the liquid, and as this deteriorates we should only obtain small supplies at a time, and I believe it is better to use but half the liquid in a bottle. Before using always stir the contents of the bottle.

We have not yet obtained possession of a plastic filling which is in all respects perfect, and if by pointing out the good as well as the bad features of the preparations that we use we can encourage manufacturers to further improve them, we may eventually have placed in our hands a filling material to our entire satisfaction.—*Dominion Dental Journal.*

